# Paracrine Diffusion of PrP^C^ and Propagation of Prion Infectivity by Plasma Membrane-Derived Microvesicles

**DOI:** 10.1371/journal.pone.0005057

**Published:** 2009-04-01

**Authors:** Vincenzo Mattei, Maria Grazia Barenco, Vincenzo Tasciotti, Tina Garofalo, Agostina Longo, Klaus Boller, Johannes Löwer, Roberta Misasi, Fabio Montrasio, Maurizio Sorice

**Affiliations:** 1 Dipartimento di Medicina Sperimentale, “Sapienza” University, Rome, Italy; 2 Laboratorio di Medicina Sperimentale e Patologia Ambientale, “Sapienza” University, Polo Universitario di Rieti “Sabina Universitas“, Rieti, Italy; 3 Prion Research Group, Paul-Ehrlich-Institut, Paul-Ehrlich-Strasse, Langen, Germany; Universidade Federal do Rio de Janeiro (UFRJ), Instituto de Biofísica da UFRJ, Brazil

## Abstract

Cellular prion protein (PrP^c^) is a physiological constituent of eukaryotic cells. The cellular pathways underlying prions spread from the sites of prions infection/peripheral replication to the central nervous system are still not elucidated. Membrane-derived microvesicles (MVs) are submicron (0.1–1 µm) particles, that are released by cells during plasma membrane shedding processes. They are usually liberated from different cell types, mainly upon activation as well as apoptosis, in this case, one of their hallmarks is the exposure of phosphatidylserine in the outer leaflet of the membrane. MVs are also characterized by the presence of adhesion molecules, MHC I molecules, as well as of membrane antigens typical of their cell of origin. Evidence exists that MVs shedding provide vehicles to transfer molecules among cells, and that MVs are important modulators of cell-to-cell communication. In this study we therefore analyzed the potential role of membrane-derived MVs in the mechanism(s) of PrP^C^ diffusion and prion infectivity transmission. We first identified PrP^C^ in association with the lipid raft components Fyn, flotillin-2, GM1 and GM3 in MVs from plasma of healthy human donors. Similar findings were found in MVs from cell culture supernatants of murine neuronal cells. Furthermore we demonstrated that PrP^Sc^ is released from infected murine neuronal cells in association with plasma membrane-derived MVs and that PrP^Sc^-bearing MVs are infectious both *in vitro* and *in vivo*. The data suggest that MVs may contribute both to the intercellular mechanism(s) of PrP^C^ diffusion and signaling as well as to the process of prion spread and neuroinvasion.

## Introduction

Prion diseases are a complex group of fatal neurodegenerative disorders that affect humans and a wide variety of animals and are characterized by strong neuronal cell loss, spongiform vacuolation and astrocytic proliferation [1]. According to the “protein-only” hypothesis PrP^Sc^, the misfolded form of normal ,0cellular prion protein (PrP^C^), is the infectious agent that may convert PrP^C^ to PrP^Sc^ in a self-propagating reaction [2]. Prions accumulate not only in the central and peripheral nervous system but also in extracerebral compartments, such as secondary lymphoid organs and muscles. However, the only organ system in which severe histopathological damage can be demonstrated as a consequence of infection with prions is the nervous system. Prion diseases are typically initiated by infection of peripheral sites, as in the case of bovine spongiform encephalopathy (BSE), variant Creutzfeldt–Jakob disease (vCJD), Kuru, and most cases of iatrogenic Creutzfeldt–Jakob disease (iCJD). The mechanisms by which prions spread from the site of peripheral exposure, such as the gastrointestinal tract, to the lymphoreticular system where a first replication phase occurs and subsequently to and within the central nervous system are still not completely elucidated [3], [4], [5]. Although different cell types of the immune system, such as B lymphocytes [6], [7], [8] follicular dendritic cells [9], [10], [11], macrophages [12], [13] and dendritic cells [14], [15], [16], [17], and the peripheral nervous system [18], [19], [20] have been recognized as key players in the process of prion neuroinvasion, relatively little information is available about the mechanism(s) underlying intercellular prion transfer.

Microvesicles (MVs) are submicron (0.1–1 µm) [21], [22], membrane-bounded vesicles which are released both from the cell surface of normal healthy or damaged cells. Shedding of membrane-derived MVs is a physiological phenomenon that usually accompanies cell activation and growth. MVs are characterized by the presence of adhesion molecules, MHC I molecules, as well as of membrane antigens typical of their cell of origin [21]–[23]. They are normal constituents of blood (5–50 µg/ml), and are secreted by leukocytes, endothelium, platelets and erythrocytes. The number of MV circulating in peripheral blood increases during cell injury, apoptosis, inflammation, thrombosis and platelet activation [23].

Although the molecular basis of protein sorting during MVs formation is not fully understood, they result from an exocytotic budding process involving lipids and proteins metabolism. The segregation of specific proteins is followed by blebbing of the membrane surface, leading to the formation of MVs and their release in the extracellular environment. Some evidence shows that MVs components mainly arise from lipid rafts, which therefore might be involved in setting up sorting platforms to concentrate specific proteins within MVs, which could be destined to extracellular secretion [24].

The functional role of MVs is still largely unknown, however recent evidence suggests that MVs are important modulators of cell-to-cell communication and play an important pleiotropic role in many biological processes. Indeed, MVs may possibly act as paracrine vectors of transcellular exchange of messages between circulating and endothelial cells [24], participate in a variety of intracellular adhesion processes, and induce cellular response(s) [23].

Exosomes, which measure about 50–90 nm in diameter, are membrane vesicles released into the extracellular environment upon exocytic fusion of multivesicular endosome with the cell surface. They have a particular composition [26] reflecting their origin in endosomes as intraluminal vesicles. It is assumed that exosomes are non-plasma-membrane-derived vesicles. Moreover, exosomes may “hijack” infectious particles such as the immuno deficiency virus (HIV) from the cytoplasm of the releasing cells [27], [28] and possibly even whole intact organelles such as the mitochondria [29].

The release of PrP^C^ and infectious PrP^Sc^ by prion infected epithelial, neuroglial and neuronal cells in association with exosomes has recently been highlighted [30]–[32], suggesting that PrP^Sc^-bearing exosomes may provide a mechanism for intercellular transmission of infectious prions in addition to cell–cell contact. It has furthermore been shown that endogenous PrP^C^ is associated with exosomes released by blood platelets [33], and a number of studies have demonstrated that prions are present in blood and blood components, buffy coats, plasma, and platelets in animal models [34], [35]. It has also been demonstrated that blood as well as plasma of animals experimentally infected with TSEs can efficiently transmit prion infection by transfusion [35]–[37]. Of concern, is the finding that vCJD is most probably efficiently transmitted between human patients by blood transfusion [38]–[40]. This infection process, in the absence of sensitive vCJD blood tests for screening of blood components donations, could lead to a horizontal spread of vCJD within the human population.

The aims of this study were to evaluate the potential role of plasma membrane-derived MVs in the mechanism(s) of PrP^C^ diffusion and prion infectivity transmission *in vitro* and *in vivo* and to characterize the interactions of PrP^C^ with lipid raft components in MVs. Here, we demonstrate that PrP^C^ is associated with MVs from plasma of human healthy donors and with MVs shed by murine neuronal cells. We further show that in MVs PrP^C^ associates with lipid raft components such as Fyn, flotillin-2, GM1 and GM3. Furthermore, we demonstrate for the first time that mouse neuronal Neuro-2a cells, endogenously expressing murine PrP, release PrP^C^ and PrP^Sc^ in association with plasma membrane-derived MVs when infected with a mouse-adapted scrapie strain. Moreover, we show that PrP^Sc^-bearing MVs can transmit prion infectivity both *in vitro* and *in vivo*, indicating that MVs contribute both to the intercellular mechanism(s) of PrP^C^ diffusion and to the process of infectious prions intercellular trafficking. We therefore postulate that MVs may act as carriers of prion infectivity during prion neuroinvasion and may also contribute to blood transfusion-mediated transmission of prion diseases.

## Materials and Methods

### Cell lines

Murine Neuro-2a cells (American Type Culture Collection ATCC CCL 131) were maintained in DMEM (Sigma, Germany) supplemented with 10% heat-inactivated fetal calf serum (FCS) (Invitrogen, Germany), 4 mM L-Glutamin (Sigma, Germany) and antibiotics (200 U/ml penicillin and 0.2 mg/ml streptomycin (Sigma, Germany) at 37°C in a humidified 5% CO_2_ atmosphere. Murine Neuro-2a PK1 cells and Rocky Mountain Laboratory strain (RML)-infected Neuro-2a PK1 cells [41] were maintained in Opti-MEM (Invitrogen, Germany) supplemented with 10% (FCS), 4 mM L-Glutamin and antibiotics (200 U/ml penicillin and 0.2 mg/ml streptomycin). The cell lines were maintained at 37°C in a humidified 5% CO_2_ atmosphere.

### Antibodies

Monoclonal anti-PrP antibody 6H4 (Prionics Switzerland). Anti-mouse immunoglobulin-colloidal gold (Biocell, UK). Monoclonal anti-PrP antibody SAF 32 (SPI Bio, Italy). Polyclonal anti-PrP antibody C-20, polyclonal anti-PrP PE-conjugated antibody (C-20 PE), polyclonal anti-Fyn antibody FYN3, polyclonal anti-Flotillin-2 antibody, polyclonal anti-Tsg101 (M-19) were purchased from Santa Cruz Biotechnology, (Santa Cruz, CA, USA). Monoclonal anti-GM2 and anti-GM3 [42] were purchased from Seikagaku (Corp. Chuo-ku,Tokyo, Japan). PE-conjugated anti-mouse IgG, HRP-conjugated cholera toxin, B subunit (HRP-CTxB), HRP-conjugated anti-mouse IgG (anti-mouse IgG-HRP), HRP anti-mouse IgM (anti-mouse IgM-HRP), HRP-conjugated anti-rabbit IgG (anti-rabbit IgG-HRP), HRP anti-goat IgG (anti-goat IgG-HRP), IgG from goat serum with irrelevant specificity (IgG irrelevant), monoclonal anti-GAP 43 antibody, were purchased from Sigma Chem. Co. (St Louis, MO, USA). B-PE-conjugated IgG from goat serum with irrelevant specificity (goat IgG B-PE irrelevant) was purchased from ICN Biomedicals (Milano, Italy). Monoclonal anti-Alix IgG_1_ 3A9 was purchased from Cell Signaling (USA) Anti-lyso-bis-phosphatidic acid (LBPA) MoAbs were kindly provided by Dr Jean Gruenberg. PE-conjugated anti-human MHC-I was purchased from eBioscience (San Diego, CA, USA).

### Preparation of platelet-free plasma samples for MVs analysis

For platelet-free plasma preparation, 5 ml blood samples were drawn by venipuncture into 0.129 M trisodium citrate. MVs were separated from whole blood within 1 h by two sequential centrifugations: 15 min at 1500 g, followed by a 1 min decantation at 13000 g to remove all the residual platelets or cell fragments of similar size [43].

Fifty µl of platelet-free plasma were labeled with 2 µg anti-PrP (C-20 PE), at +4°C. The fluorescence intensity was analyzed with a IL Coulter cytometer (Coulter Electronics, Hialeah, FL) and compared with a sample of platelet-free plasma, labeled with goat IgG B-PE irrelevant. The size distribution of MVs was 0.1 to 1 µm; MVs were gated on the basis of forward angle light scatter and 90° light scatter parameters, using polystirene latex beads (Sigma, St Louis, MO, USA) 0.8 µm as standard. To distinguish eventual residual platelets in our sample, we compared the MVs preparation with a platelet-enriched plasma, stained by anti-CD41 MoAb (data not show).

Separately, in parallel experiments MVs, collected by centrifugation at 20000×g, were resuspended in 50 µl of sample buffer and analyzed by Western Blot [44], using the anti-PrP 6H4, and then anti-mouse IgG-HRP, anti-Fyn (FYN3) and then anti-rabbit IgG-HRP, or anti-flotillin-2 and then anti-goat IgG-HRP. Immunoreactivity was assessed by chemiluminescence reaction using the ECL Western blocking detection system (Amersham Biosciences). As a control, cells were separated from human peripheral blood (PB) by centrifugation and resuspended in cold lysis buffer [20 mM Tris-HCl (pH 7.5), 1 mM EDTA, 100 mM NaCl, 1% Triton X-100, 0.5% sodium deoxycholate, 0.5% SDS]. Lysates were pelleted at 20000×g for 10 min at 4°C and supernatants were transferred to a clean microfuge tube. Protein concentration for cell lysates and MVs was determined by Bradford assay as recommended by the manufacturer's guidelines (Roth, Germany).

### Isolation of MVs from cell culture supernatants

Neuro-2a or Neuro-2a PK1 cells (2–4×10^7^ cells) were cultured for 2–3 days prior to MVs isolation by sequential centrifugation protocol [45], [46]. Cellular debris was removed by two consecutive centrifugation steps at 4500×g for 5 min at RT. Supernatants were centrifuged at 20000×g for 1 h at 4°C. MVs were pooled, washed in either PBS or Opti-MEM (Invitrogen), repelleted and then either resuspended in Opti-MEM for *in vitro* or in PBS (Invitrogen, Germany) for *in vivo* infection experiments or lysed [20 mM Tris-HCl (pH 7.5), 1 mM EDTA, 100 mM NaCl, 1% Triton X-100, 0.5% sodium deoxycholate, 0.5% SDS] for immunoblot analyses.

### Immunoelectron microscopy

Neuro-2a PK1 cells were incubated with the anti-PrP 6H4 antibody for 1 h at 4°C. Cells were washed in PBS and then were fixed with 1% paraformaldehyde for 1 h at 4°C, washed and labeled with anti-mouse immunoglobulin-colloidal gold for 3 h at 4°C. Cells were post-fixed first in 2.5% glutaraldeyde for 45 min at RT and then in osmium tetroxide 1% in Veronal acetate buffer, pH 7.4, for 2 h at 4°C, stained with uranyl acetate (5 mg/ml), dehydrated in acetone and embedded in Epon 812. Samples were examined under an electron microscope (Zeiss, Germany).

### Western blot analysis

Eighty micrograms of total protein of each cell lysate and 20 µg of each MVs were electrophoresed through a 12% SDS polyacrylamide gel. When proteinase K digestion was performed prior to immunoblot analysis, 250 µg of cell lysates and 20 µg of MVs preparations were digested for 1 h at 37°C (0.1 µg or 0.3 µg or 1 µg PK (Roche Diagnostics NL, Germany) per 20 µg total protein). Proteinase K digestion was stopped with 1 mM phenylmethylsulphonyl fluoride (PMSF, Sigma, Germany). Proteins were transferred to PVDF membranes (Immobilon-P; Millipore, USA) by semidry blotting. Membranes were blocked at RT for 1 h with Tris-buffered saline/0.05% Tween 20 (TBST)/5% nonfat dry milk, incubated with the appropriate primary antibody diluted in Tris-buffered saline/0.05% Tween 20 (TBST)/1% nonfat dry milk (for PrP, anti-PrP SAF-32 or anti-PrP 6H4; anti-GAP-43; anti-flotillin-2; anti-Fyn FYN3;, anti-Alix 3A9; anti-Tsg101 (M-19), overnight at 4°C. After washing with TBST, membranes were incubated for 1 h at room temperature to horseradish peroxidase-conjugated secondary antibody diluted in the same buffer as above. Bands were visualized by enhanced chemiluminescence (Amersham Pharmacia, Germany).

### Immunoprecipitation experiments

Briefly, blood plasma-derived and Neuro-2a cells-derived MVs were lysed in lysis buffer (20 mM HEPES, pH 7.2, 1% Nonidet P-40, 10% glycerol, 50 mM NaF, including protease inhibitors). After preclearing, the supernatant was immunoprecipitated with anti-PrP polyclonal antibody C-20 plus protein A-acrylic beads. The immunoprecipitates were split into two aliquots.

The first one was subjected to ganglioside extraction according to the method of Svennerholm and Fredman [47]. The eluted glycosphingolipids were dried and separated by high-performance-thin-layer-chromatography (HPTLC) aluminium-backed silica gel 60 (20×20) plates (Merck, Darmstadt, Germany). Chromatography was performed in chloroform∶methanol∶0.25% aqueous KCl (5∶4∶1) (v∶v∶v). Plates were immunostained for 1 h at room temperature with HRP-CTxB, or, alternatively, with anti-GM2 IgM or anti-GM3 IgM and then with anti-mouse IgM-HRP. Immunoreactivity was assessed by chemiluminescence reaction using the ECL Western blocking detection system (Amersham, Buckinghamshire, UK). Alternatively, the immunoprecipitates were subjet to Western Blot analysis by anti-Fyn FYN3 and then with anti-rabbit IgG-HRP.

The immunoprecipitates were checked by Western blot, using the anti-PrP monoclonal 6H4 antibody.

### *In vitro* infectivity assay

Noninfected Neuro-2a PK1 cells were seeded in six-well plates 24 hrs prior to infection. MVs were isolated both from uninfected (MVs^Noninf^) and RML-infected (MVs^Inf^) Neuro-2a PK1 cells as previously described and resuspended in Opti-MEM. MVs were incubated for 48 hrs with noninfected Neuro-2a PK1 cells. RML and mock brain homogenates (1% w/v homogenates) were included as positive and negative controls, respectively. Prion infection was assayed by the presence of PrP^Sc^ by cell blot assay.

### Cell blot assay

Cells were cultured on glass coverslips (Roth, Germany) until confluence, washed with CMF-PBS, and placed cell side down on lysis buffer I [50 mM Tris-Cl, pH 8.0; 150 mM NaCl; 0.5% sodium deoxycholate; 0.5% Triton X-100]-soaked nitrocellulose membranes (Hybond-C-Extra, Amersham, Germany) that were previously laid on a lysis buffer-soaked filter paper. Membranes were then processed as described [41]. Briefly, membranes were dried at RT, coverslips were carefully removed and then the membranes were incubated in a Proteinase K solution [5 µg/ml PK in lysis buffer] for 90 min at 37°C. After twice wash in dH_2_0, PK digestion was stopped by incubating the membranes in 2 mM PMSF for 10 min at RT. Membranes were than treated with 3 M guanidinium thiocyanate in 10 mM Tris-Cl (pH 8.0) for 10 min. After extensive washing in dH_2_0, membranes were processed for PrP^C^/PrP^Sc^ detection by the use of the anti-PrP 6H4 as described above.

### *In vivo* infectivity bioassay

MVs were isolated both from uninfected and RML-infected Neuro-2a PK1 cells, as previously described, and resuspended in PBS, 0.2 ml. Uninfected and infected Neuro-2a PK1 cells were collected and resuspended in 1 ml PBS. MVs and cells were subjected to five consecutive cycles of freeze and thawing. Protein concentration for cell lysates and MVs was determined by Bradford assay, as recommended by the manufacturer's guidelines. Samples were then adjusted to 10 µg total protein per 30 µl with PBS/5%BSA. Different dilutions of mouse brain homogenate infected with the Rocky Mountain Laboratory (RML) scrapie strain (passage 5.0, 1×10^8^ LD_50_/ml 10% brain homogenate) [48] in PBS/5% BSA were used as positive controls, whereas mock infected brain homogenate was included as negative control.

Thirty microliters for each sample were administered intracerebrally to groups of four *t*g*a20* mice [49]. Disease in animals was diagnosed when at least three of the following symptoms were observed: foot clasping of hindlegs when mice were lifted by the tail, plastic tail; decreased motor activity; mincing gait, disorientation; mild hind leg paresis, ataxia; kyphosis. Incubation time to terminal scrapie sickness was determined and infectivity titers were calculated by using the relationship *y* = 11.45−0.088 *x*, where *y* is logLD_50_/ml homogenate and *x* is incubation time in days to terminal disease, was used to calculate infectivity titres. The presence of a protease-resistant isoform of PrP (PrP^Sc^) in the infected brains was investigated on proteinase K-treated (20 µg/ml; 30 min; 37°C) homogenates by Western blot analysis, as described above.

## Results

### Presence and association of PrP^C^ and lipid raft components in human plasma-derived microvesicles

**Figure 1:**
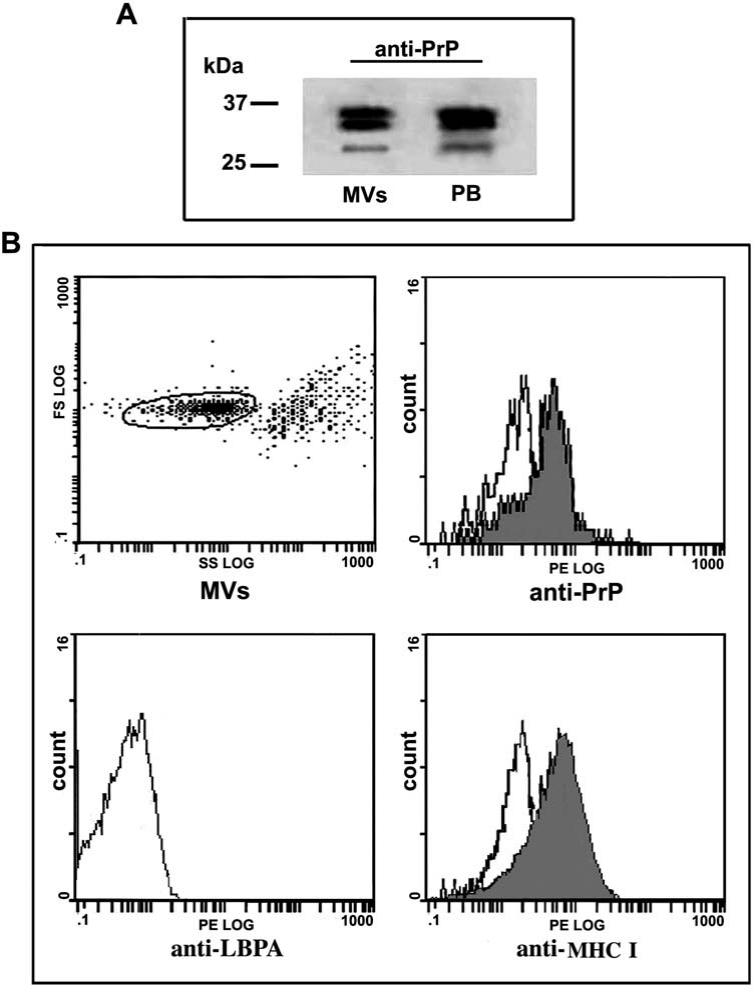
PrP^C^ detection in MVs from human plasma. MVs obtained from human plasma were analyzed for the presence of PrP^C^. A: Western blot analysis with anti-PrP monoclonal 6H4 antibody of MVs compared with human peripheral blood (PB). B: Cytofluorimetric analysis with an anti-PrP (C-20 PE). MVs: MVs gated on the basis of forward and side scatter parameters using polystirene beads 0.8 µm as standard. Anti-PrP: MVs stained by anti-PrP PE (C-20 PE) vs IgG PE with irrelevant specificity. Anti-LBPA: MVs stained by anti-LBPA, followed by PE-conjugated anti-mouse IgG. Anti-MHC-I: MVs stained by anti-human MHC I vs IgG PE with irrelevant specificity.

MVs are normal constituents of blood plasma and are secreted by leukocytes, erythrocytes, platelets and endothelium [50]. To investigate the presence of PrP^C^ in cell membrane-derived MVs from plasma obtained from healthy human peripheral blood donors, MVs were isolated by sequential centrifugations from platelet-free plasma preparations. Presence of PrP^C^ in the blood plasma-derived MVs was first detected by immunoblot analysis by the use of the monoclonal anti-PrP 6H4 antibody; PrP^C^ was recognized such as shown in Figure 1A. As shown in Figure 1B, PrP^C^ expression in MVs was confirmed by flow cytometry: staining with the anti-PrP polyclonal antibody C-20 PE revealed a specific PrP^C^ reactivity. These findings indicate the presence of PrP^C^ on MVs isolated from human plasma. In order to check the purity of MVs under the chosen test condition, two control stainings were performed: (i) anti-LBPA, which is an endosome-specific marker [51], thus representing an useful tool to identify exosomes, but not MVs; (ii) anti-MHC I as a positive control for MVs. As expected, MVs were virtually negative for anti-LBPA staining, demonstrating the absence of exosomes in our preparation. However, a specific anti-MHC I staining was detected, confirming both the isolation of MVs and the purity of the MV preparation (Fig. 1).

**Figure 2:**
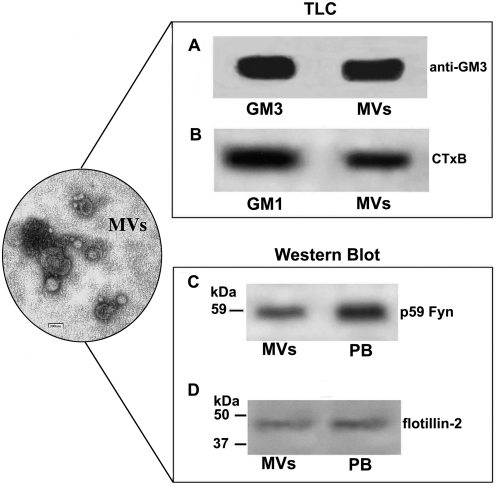
Evidence for raft components in MVs from human plasma. MVs obtained from human plasma were subjected to ganglioside extraction and analyzed by TLC immunostaining. A: reactivity of anti-GM3, followed by peroxidase-conjugated anti-mouse IgM MoAb; B: reactivity of HRP-CTxB. MVs obtained from human plasma were analyzed by Western blot using the anti-Fyn (FYN3) (C) and anti-flotillin-2 (D). Inset: electron microscopy negative staining of MVs obtained from human plasma.

Having previously demonstrated that PrP^C^ is strongly associated with gangliosides GM1 and GM3 within lipid rafts on the surface of lymphocytic cells [52], [53], we investigated the presence of GM1 and GM3 in MVs from plasma of human healthy donors. Acidic glycosphingolipids extracted from MVs were immunostained by anti-GM3 antibody (MoAb GMR6) [42] or by HRP-CtxB, which stains GM1 [54]. As shown in Figure 2, the presence of GM3 (Fig. 2A) and GM1 (Fig. 2B) in isolated MVs was clearly detected. Since p59Fyn kinase as well as flotillin-2 have also been indicated as raft components, which may be associated with PrP^C^ [53], [55], [56], [57], we then analyzed the presence of both proteins in blood plasma-derived MVs. Western blot analyses showed a 59 kDa band specifically recognized by the anti-Fyn antibody (Fig. 2C) and a 42 kDa band specifically recognized by the anti-flotillin-2 antibody (Fig. 2D). The purity and enrichment of MVs was shown by electron microscopy (Fig. 2E).

**Figure 3:**
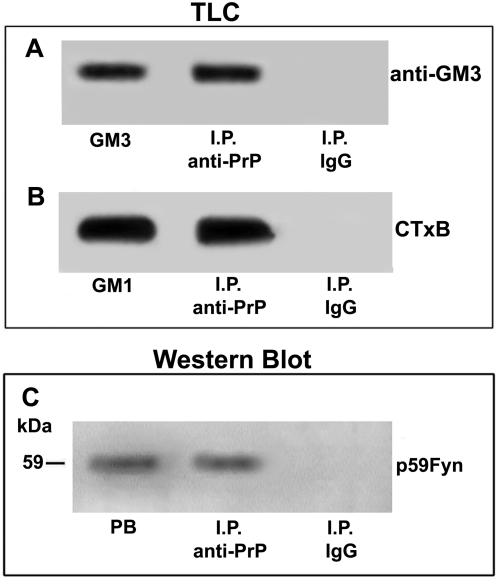
Co-immunoprecipitation of PrP^C^ with gangliosides and p59 Fyn Kinase. MVs from human plasma were immunoprecipitated with anti-PrP (C-20). The immunoprecipitates were subjected to ganglioside extraction and analyzed by TLC immunostaining or Western Blot with polyclonal anti-Fyn antibody (FYN3). A: reactivity of anti-GM3 (MoAb GMR6) with standard GM3, PrP^C^ immunoprecipitate and immunoprecipitate with IgG with irrelevant specificity. B: reactivity of CTxB perox with standard GM1, PrP^C^ immunoprecipitate and immunoprecipitate with IgG with irrelevant specificity. C: Western blot analysis with anti-Fyn (FYN3) of human peripheral blood, PrP^C^ immunoprecipitate and immunoprecipitate with IgG with irrelevant specificity.

Direct interaction of PrP^C^ with gangliosides as well as with p59Fyn kinase in plasma-derived MVs was assayed by coimmunoprecipitation followed either by TLC immunostaining for the detection of gangliosides or conventional immunoblotting for p59Fyn kinase detection. As shown in Figure 3, PrP^C^ associates in MVs with the gangliosides GM3 (Fig. 3A) and GM1 (Fig. 3B) and with the tyrosine kinase Fyn (Fig. 3C). These findings suggest that raft components, strictly associated with PrP^C^ on the cell plasma membrane, such as gangliosides, p59Fyn and flotillin-2, are recruited during membrane blebbing which leads to MVs formation.

### Neuronal cells release membrane-derived MVs bearing PrP^C^ and raft components

**Figure 4:**
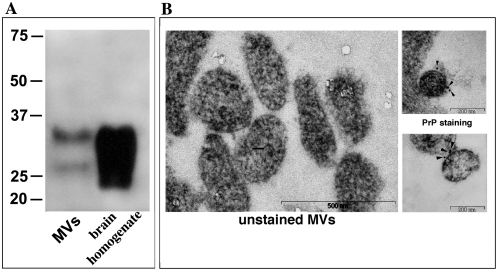
Evidence for the presence of PrP^C^ in MVs from neuronal cells. A MVs obtained from the Neuro-2a cells were analyzed by Western blot using anti-PrP SAF-32. Crude brain homogenate from wild type mice was included as positive control. B Left: electron microscopy negative staining of MVs obtained from Neuro-2a PK1 cells. Right: two representative examples of anti-PrP^C^ specific staining on the surface of MVs obtained from Neuro-2a PK1 cells. PrP^C^ was revealed by immunogold labelling (10 nm) and indicated on the images by the black arrows.

In order to determine whether neuronal cells can also release plasma membrane-derived MVs and to investigate whether these MVs also contain PrP^C^ associated with lipid rafts components, we isolated MVs from cell culture supernatants of a murine neuronal cell line (Neuro-2a) and from its subclonal line (Neuro-2a PK1) [41]. The isolation of MVs from cell culture supernatants was based on a sequential centrifugation steps protocol in which increasing centrifugal forces were used. Plasma membrane-derived MVs were collected at 20000×g, a centrifugal force not sufficient to enrich for exosomes. The presence of PrP^C^ was shown in MVs released both by Neuro-2a (Fig. 4A) and Neuro-2a PK1 cells. Electron microscopy analyses demonstrated the presence of vesicles, often aggregated, with a size (from 100 nm to 1 µm) compatible with MVs similarly purified from other cells (Fig. 4B) [23]. In particular, immunogold labeling of Neuro-2a PK1 cells revealed that PrP^C^ is unevenly distributed on the membrane of MVs (Fig. 4B).

To exclude the presence of contaminating exosomes within the MVs-enriched fraction, the levels of Tsg101 and Alix, both cytoplasmic proteins previously identified as specific markers for exosomes [46], [58], were assessed by immunoblot analysis (Fig. S1). The signals obtained in the MVs-enriched fraction were lower than those observed in the exosome-enriched fraction.

**Figure 5:**
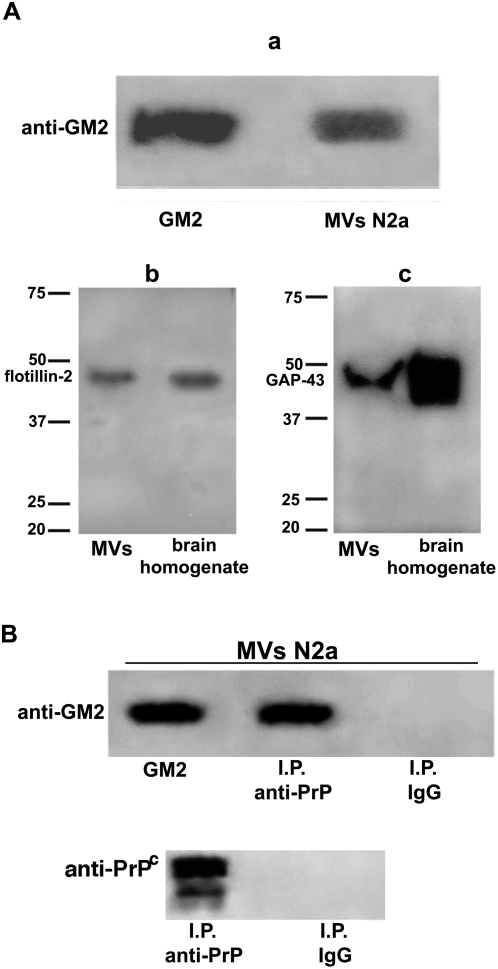
Evidence for raft components in MVs from neuronal cells. A MVs from Neuro-2a cells were subjected to ganglioside extraction and analyzed by TLC immunostaining. a: reactivity of anti-GM2 (GMR6 MoAb) with standard GM2. MVs obtained from the Neuro-2a cells were analyzed by Western blot using different antibodies. b: reactivity with anti-Flotillin-2; c: reactivity with anti-GAP-43. Crude brain homogenate from wild type mice was included as positive control. B Co-immunoprecipitation of PrP^C^ and gangliosides. MVs from Neuro-2a cells were immunoprecipitated with anti-PrP (C-20). The immunoprecipitates were subjected to ganglioside extraction and analyzed by TLC immunostaining: reactivity of anti-GM2 (GMR6 MoAb) with standard GM2, PrP^C^ immunoprecipitate and immunoprecipitate with IgG with irrelevant specificity. The immunoprecipitate was revealed as PrP^C^, as detected by Western blot, using the anti-PrP monoclonal 6H4 antibody.

Since we have shown that blood plasma-derived MVs contain lipid raft components, such as Flotillin-2, gangliosides and, most importantly, PrP^C^, we analyzed the presence of these components in MVs released by Neuro-2a cells by Thin Layer Chromatography (TLC) immunostaining and immunoblot analyses (Fig. 5). Since the main ganglioside constituent of Neuro-2a was GM2 [59], acidic glycosphingolipids extracted from MVs were immunostained with a highly specific anti-GM2 antibody. As shown in Figure 5A, the presence of GM2 in isolated MVs was detected. In addition, neuronal cells-derived MVs also contained Flotillin-2 and the axonal membrane protein GAP-43, a neuronal protein that binds to rafts via its acylated N-terminal.

Since gangliosides have been shown to be components of the signaling complex within lipid rafts [60], we investigated the association of PrP^c^ with ganglioside GM2 in MVs from Neuro-2a. MVs were isolated by sequential centrifugations as above and PrP^C^ was immunoprecipitated with the anti-PrP polyclonal antibody C-20. Acidic glycosphingolipids extracted from the PrP^C^ immunoprecipitates were analyzed by Thin Layer Chromatography (TLC) immunostaining, using the anti-GM2 MoAb. The results indicated that PrP^C^ associates with GM2 (Fig. 5B) within MVs rafts. The immunoprecipitate was revealed as PrP^C^, as detected by Western blot, using the anti-PrP monoclonal 6H4 antibody. In control samples the immunoprecipitation using IgG with irrelevant specificity, under the same condition, did not result in detectable levels of gangliosides.

These findings clearly indicate that neuronal cells actively release membrane-derived MVs in addition to exosomes [61]. The secreted MVs contain lipid raft components, suggesting that also in the CNS MVs-based intercellular transmission of molecular information could occur.

### MVs secreted by neuronal cells harbour both PrP^Sc^ and prion infectivity

**Figure 6:**
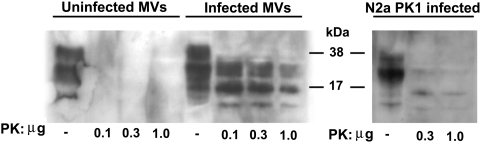
Prion infected neuronal cells shed PrP^Sc^ bearing MVs. Left panel: Detection of PrP^Sc^ in MVs isolated from culture supernatants of prion uninfected and infected Neuro-2a PK1 cells. Twenty µg total proteins of isolated MVs were PK digested. PrP^Sc^ was specifically detected in MVs released by the infected but not from the uninfected N2a PK1 cells. Right panel: Detection of PrP^Sc^ in infected Neuro-2a PK1 cells. Two hundred and fifty µg total proteins of cell lysates were PK digested. The presence of PK-resistant PrP^Sc^ was assessed with increasing amounts of proteinase K and detected by the use of the 6H4 antibody. The positions of the molecular weight standards (in kilodaltons) are indicated.

In order to assess whether prion infected neuronal cells may release membrane-derived MVs that harbor the pathological isoform of the prion protein (PrP^Sc^) and prion infectivity, we isolated MVs from cell culture supernatants of Rocky Mountain Laboratory prion strain (RML)-infected and non-infected Neuro-2a PK1 cells. We then assessed the presence of PrP^Sc^ by immunoblot analysis and that of prion infectivity by *in vitro* and *in vivo* infectivity transmission experiments. Specific detection of PrP^Sc^ was achieved by the use of proteinase K (PK) digestion. The abnormally folded PrP^Sc^ is partially resistant against PK treatment, whereas the cellular isoform PrP^C^ is completely degraded by this protease [62]. As shown in Figure 6, MVs derived from infected cells harbored PK-resistant PrP, a clear indication for the presence of PrP^Sc^. Interestingly, the levels of PrP^Sc^ detected in the purified MVs were higher than those observed in the parental cell line (Fig. 6), suggesting that PrP^Sc^ is enriched on the membrane of MVs. PrP^Sc^ was not detected, as expected, in MVs secreted by non-infected Neuro-2a PK1 cells (Fig. 6), confirming that PrP^Sc^ is released specifically from prion infected neuronal cells.

**Figure 7:**
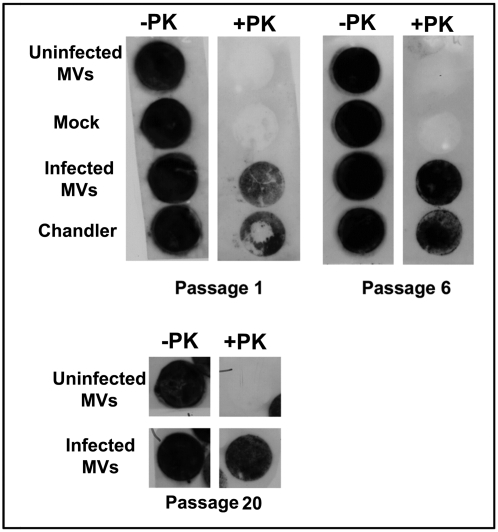
*In vitro* transmission of prion infectivity by PrP^Sc^-bearing MVs. Cell blot assay detecting *de novo* infected N2a PK1 cells at serial culture passages. N2a PK1 cells were incubated with MVs isolated from uninfected (Mvs^Uninf^) or from infected (MVs^Inf^) N2a PK1 cells. Mock and Chandler infections were also included as negative and positive control, respectively. A time-dependent increase of PrP^Sc^ levels was detected after proteinase K treatment (+PK).

MVs-dependent transmission of prion infectivity was then first assayed by an *in vitro* infection approach. Noninfected Neuro-2a PK1 cells were incubated in the presence of MVs isolated from cell culture supernatants of infected and noninfected Neuro-2a PK1 cells, respectively. *De novo* amplification of PrP^Sc^ was then followed for longer than 3 months post infection by cell blot assay (Fig. 7). Conversion of the endogenous PrP^C^ into the prion disease-associated pathological isoform PrP^Sc^ was only detected in Neuro-2a PK1 cells treated with MVs derived from prion-infected but not from non-infected donor cells (Fig. 7). Prion replication was detected up to 30 passages after infection, indicating that MVs-dependent prion transmission generate a persistent infection in the recipient neuroblastoma cells and excluding that the detected PrP^Sc^ could just be due to the persistence of the initial inoculum.

In order to further confirm the association of prion infectivity with membrane-derived MVs and to determine prion titres, *in vivo* biossay was performed in which purified MVs from infected and non-infected Neuro-2a PK1 cells or cell lysates were intracerebrally inoculated into *tg*a*20* indicator mice [49]. Incubation times until development of terminal scrapie were determined and infectious titres were calculated by comparing incubation times against a calibration curve (Table 1). Indicator mice developed clinical prion disease after inoculation with Rocky Mountain Laboratory mouse-adapted prion strain (RML) at the three dilutions tested, but not when they were inoculated with mock control, as expected. Interestingly, membrane-derived MVs from infected but not from non-infected Neuro-2a cells were capable of transmitting prion disease to *tga20* indicator mice, demonstrating that MVs are infectious not only *in vitro* but also *in vivo*. Furthermore, MVs-associated prion titres were higher than those detected as cell-associated (Table 1), a finding confirming the observation that MVs contain more PrP^Sc^ than the cells from which they are derived (Fig. 6). On the other hand, MVs seem to be a little less infectious than crude brain homogenate: indeed mice challenged with 6×10^−2^ dilution of RML (i.e. corresponding to 10 µg of total brain protein) developed clinical prion disease earlier than those challenged with MVs.

**Table 1:** PrP^Sc^-bearing MVs transmit disease to *Tga20* indicator mice.

Inoculum	Indicator mice succumbing to scrapie	Scrapie death Days (mean+/−STDEV)	Estimated infectivity titres (log LD_50_/ml)
Mock 10^−4^	0/4	>150	<1.5
RML 10^−4^	4/4	78±2	4.6
RML 10^−2^	4/4	68±2	5.5
RML 6×10^−2^	4/4	64±2	5.8
MVs^Noninf^	0/4	>150	<1.5
MVs^Inf^	4/4	68±2	5.5
Neuro-2a PK1 cells	0/4	>150	<1.5
Neuro-2 a PK1 inf. cells	4/4	76±6	4.8

*Tga20* indicator mice were inoculated intracerebrally with either 30 µl (10 µg total proteins) of cell lysates or MVs or with 30 µl of different RML brain homogenate dilutions. Mock-inoculated animals were included as negative controls. Incubation time until development of terminal scrapie was determinated. The relationship *y* = 11.45−0.088 *x*, where *y* is logLD_50_/ml homogenate and *x* is incubation time in days to terminal disease, was used to calculate infectivity titres.

## Discussion

MVs are normal constituents of blood plasma and are secreted by leukocytes, endothelium, platelets and erythrocytes [50]. Interestingly, only one-third of the PrP^C^ in human blood is cell associated, and the remaining two-third is present in plasma [63]. MVs are shed from the plasma membrane of eukaryotic cells, mainly from those undergoing activation or apoptosis [22]. In this context, it has previously been shown that thrombin stimulation triggered a transient shedding of microvesicles from the platelet surface. Of note, PrP^C^ is released initially in small quantities on microvesicles and subsequently in higher levels on exosomes [33].

In the present study we demonstrated that PrP^c^ is present in MVs from human plasma of healthy donors and that, among MVs, PrP^c^ is associated with other lipid raft components. The functional role of MVs is still largely unknown, however, some evidence show that MVs components mainly arise from lipid rafts, which therefore might be involved in setting up sorting platforms to concentrate specific proteins within MVs, that could be destined to extracellular secretion. It suggests that they may participate in a variety of intercellular adhesion processes and induce cellular responses [24]. Moreover, it has been demonstrated that blood as well as plasma of animals experimentally infected with TSEs can transmit TSE infection by transfusion [35], [37]. Our results support and extend these findings, showing the presence of PrP^C^ in MVs from human plasma, as revealed by Western blot and cytofluorimetric analysis results, and demonstrating that the whole signaling complex is represented in these microparticles. Indeed, in MVs, not only PrP^C^, but also p59Fyn, as well as GM1 and GM3 gangliosides were revealed and co-immunoprecipitated with PrP^C^, demonstrating that all the signaling complex may be shed within MVs. The presence of lipid rafts-associated signaling complex(es) in MVs suggests a potential role for these microparticles in cell-to-cell communication through MVs shedding and fusion.

It has previously been shown that neuronal cells release exosomes containing numerous proteins and lipids similar to those present in the membranes of the cells from which they originate, including typical neuronal proteins such as the GluR2/3 subunits of glutamate receptors, the cell adhesion molecule L1 and, interestingly, PrP^C^ [61]. Here, we report for the first time that neuronal cells also release plasma-derived MVs. Indeed, MVs were isolated from the cell culture supernatants of two murine neuronal cell lines (Neuro-2a and Neuro-2a PK1 cells). Electron microscopy analyses of MVs preparations demonstrated the presence of vesicles with a diameter ranging from 100 nm to 1 µm, a size compatible with that described for MVs purified from other cells [23].

In this study, the association of PrP^C^ with MVs shed by murine neuronal cells was demonstrated. The presence of PrP^C^ in MVs released from both the Neuro-2a and the Neuro-2a PK1 cell lines was shown by immunoblot analysis. Furthermore, IEM analysis of Neuro-2a PK1 cells, a murine neuroblastoma cell line endogenously expressing PrP^C^, revealed the unevenly distribution of PrP^C^ on the membrane of MVs, as well as on the cell plasma membrane. Heterogeneity of PrP^C^ molecules is attributed mainly to various degrees of N-glycosylation on asparagine residues [64], [65]. Interestingly, MV-associated PrP^C^ displayed an electrophoretic mobility pattern on SDS-polyacrylamide gels resembling if not overlapping the one detected for cell-derived PrP^C^, suggesting that in MVs fully processed and mature PrP^C^ molecules are incorporated during membrane shedding. However, in a recently published work, a novel processing pathway that involves the N-terminal modification of PrP^C^ and the selection of distinct PrP^C^ glycoforms for incorporation into exosomes has been described [31]. Thus, a more accurate and detailed analysis of MV-associated PrP^C^ should be performed in order to verify whether a similar processing pathway also occurs during incorporation of PrP^C^ into plasma-derived MVs. The presence of lipid raft components, such as the p59Fyn kinase, flotillin-2 and the ganglioside GM2 was furthermore detected in MVs released by the murine Neuro-2a cell line, suggesting that raft components strictly associated with PrP^C^ on the cell plasma membrane are recruited during the formation of MVs in neuronal cells. The neuronal origin of the analyzed MVs was confirmed by the detection of the axonal membrane protein GAP-43, a neuronal protein which binds to rafts via its acylated N-termini.

The growing interest in the presence of PrP^C^ in MVs is due to the potential role of these microparticles in the propagation of the disease. The release of PrP^C^ and infectious PrP^Sc^ by prion infected epithelial (Rov cells), neuroglial (Mov cells) and neuronal cells (GT1-7 cells) in association with exosomes has recently been highlighted [30], [31], suggesting that PrP^Sc^-bearing exosomes may provide a mechanism for intercellular transmission of infectious prions in addition to cell-to-cell contact. Indeed it was found that both PrP^Sc^ and PrP^C^ are present in cell culture supernatants in a secreted, exosome-associated form and that exosomes bearing PrP^Sc^ were infectious both *in vitro* and *in vivo* [30], [31].

This work further extends these findings showing for the first time that stably infected neuronal cells endogenously expressing PrP^C^ shed plasma-derived MVs, which are carriers both of the pathological isoform PrP^Sc^ and of prion infectivity. Noteworthy, MVs isolated from cell culture supernatants of Rocky Mountain Laboratory prion strain (RML)-infected Neuro-2a PK1 harbored PK-resistant PrP, an indication for the presence of PrP^Sc^. Interestingly, the levels of PrP^Sc^ detected in the purified MVs were higher than those observed in the parental cell line, suggesting that PrP^Sc^ is enriched on the membrane of MVs. PrP^C^ and PrP^Sc^ are GPI-anchored proteins known to partition into lipid rafts [66], [67] and the lipid raft-like nature of MVs membranes argues for an efficient insertion of PrP^Sc^ in lipid raft-rich domains of the plasma membrane involved in MVs formation. Furthermore, the evidence that lipid rafts play a role in the formation of PrP^Sc^ in scrapie-infected culture cells [68], [69] supports the hypothesis of a preferential distribution of PrP^Sc^ within lipid rafts.

On the other hand, PrP^C^ but not PrP^Sc^ was detected in MVs secreted by non-infected Neuro-2a PK1 cells, confirming that PrP^Sc^ is released specifically from prion infected neuronal cells. Thus, these findings clearly indicate that prion infected neuronal cells such as the Neuro-2a PK1 shed plasma membrane-derived MVs containing PrP^Sc^.

The potential role of plasma membrane-derived MVs in prion infectivity transmission was tested both *in vitro* and *in vivo* infection models. This study demonstrates that PrP^Sc^-bearing MVs can transmit prion infectivity both *in vitro* and *in vivo*, indicating that MVs contribute both to the intercellular mechanism(s) of PrP^C^ diffusion and to the process of infectious prions intercellular trafficking. Indeed, MVs isolated from cell culture supernatants of infected but not from uninfected Neuro-2a PK1 cells were capable of initiating *de novo* amplification of prions in the recipient cells. Interestingly, prion replication was detected up to 30 passages after infection, indicating that MV-dependent prion transmission generated a persistent infection in the recipient neuroblastoma cells. Hypothetically, PrP^C^ conversion may be initiated as a consequence of the binding of PrP^Sc^-bearing MVs to acceptor cells. In this context, it is noteworthy that the topology of exosomal and, most probably, of MVs membranes is identical to that of the plasma membrane [70]. Alternatively, but not mutually exclusive, MVs captured by the target cells could fuse with the cell surface or be internalized by an unclear mode of entry to induce conformational change of PrP^C^ at the cell surface and/or in endocytic compartments, respectively. Moreover, *in vivo* biossay in transgenic mice that overexpress murine PrP^C^ and are highly susceptible to murine prions [50] clearly demonstrated that prion infectivity is associated with PrP^Sc^-harboring MVs shed by prion infected murine neuronal cells. In fact, membrane-derived MVs from infected but not those from uninfected Neuro-2a PK1 cells were capable of transmitting prion disease to *tga20* indicator mice. These new findings suggest that plasma membrane-derived MVs shed by prion infected cells could actively participate in the intercellular trafficking of PrP^Sc^ and contribute to the transmission of infectious prions. One may therefore postulate that MVs act as carriers of prion infectivity during prion neuroinvasion and play a pivotal role in the propagation of prion infectivity from neuron to neuron, in this way contributing to the pathogenesis of the disease. However, it does not exclude the possibility that also exosomes may play an important role in secreted infectivity from the cells.

In conclusion, our data provide here evidence for the presence of PrP^C^ in MVs *in vivo*, in human plasma, suggesting that these MVs might be involved in the mechanism(s) of PrP^C^ paracrine as well as endocrine diffusion and may also contribute to blood transfusion-mediated transmission of prion diseases.

## Supporting Information

Figure S1 (0.15 MB TIF)
