# Supplementary figures and images for: Paracrine Diffusion of PrPC and Propagation of Prion Infectivity by Plasma Membrane-Derived Microvesicles
Source: PLoS One. 2009 Apr 1;4(4):e5057. doi: 10.1371/journal.pone.0005057 (PMC2659799; doi:10.1371/journal.pone.0005057)

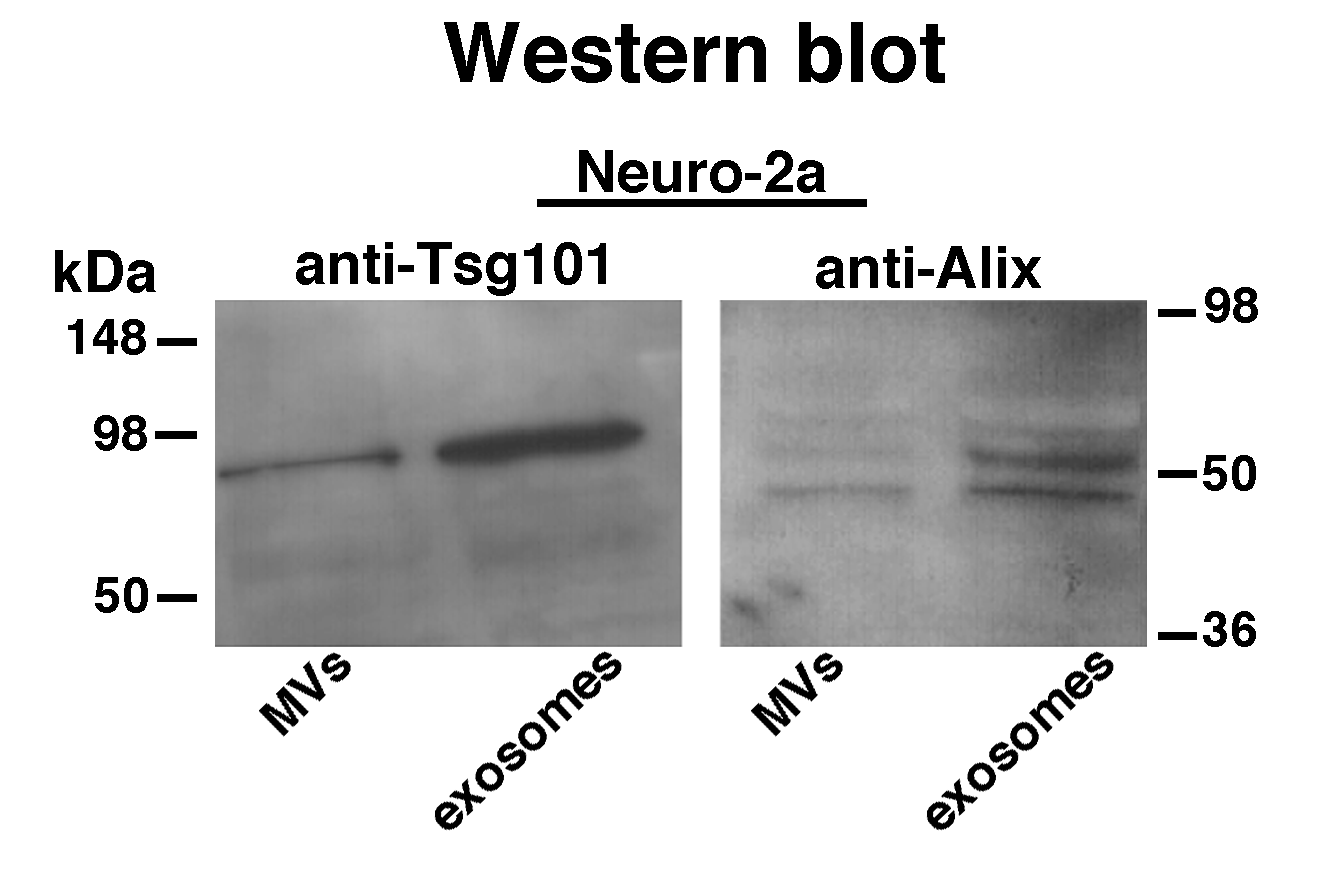

Supplement: Figure S1 — (0.15 MB TIF) [file pone.0005057.s001.tif]
